# Protective effects of orange (*Citrus sinensis L.)* peel aqueous extract and hesperidin on oxidative stress and peptic ulcer induced by alcohol in rat

**DOI:** 10.1186/s12944-017-0546-y

**Published:** 2017-08-14

**Authors:** Slimen Selmi, Kais Rtibi, Dhekra Grami, Hichem Sebai, Lamjed Marzouki

**Affiliations:** Higher Institute of Biotechnology of Beja, Laboratory Functional Physiology and Bio-resources Valorisation, University of Jendouba, Avenue Habib Bourguiba, BP, 382, 9000 Beja, Tunisia

**Keywords:** *Citrus sinensis* peel, Hesperidin, Gastric ulcer, Oxidative stress markers, COX-2, TNF-α, Rat

## Abstract

**Background:**

Massive alcohol drinking can lead to gastric ulcer. In the present study we investigated the gastroprotective effect of *Citrus sinensis* peel aqueous extract (CSPE) and Hesperidin (H) in ethanol (EtOH) induced oxidative stress and peptic ulcer in rats.

**Methods:**

Seventy adult male Wistar rats were divided into seven groups of 10 each: control, EtOH (4 g/kg b.w.), EtOH + various doses of CSPE (100, 200 and 400 mg/kg, b.w.), EtOH + Hesperidin (50 mg/kg, p.o.) and EtOH + Omeprazole (OM, 20 mg/kg, p.o.). Animals were perorally (p.o.) pre-treated with CSPE during 15 days and intoxicated with a single oral administration of EtOH (4 g/kg b.w.) during 2 h. Gastric ulcer was induced in rats with a single dose of ethanol (EtOH). Ulcer index, gene expression of gastric cyclooxygenase-2 (COX-2), tumor necrosis factor alpha (TNF-α), malondialdhyde (MDA), hydrogen peroxide H_2_O_2_ and Thiol groups (−SH) content in stomach and antioxidant enzymes superoxide dismutase (SOD), catalase (CAT) and gluthation peroxidise (GPx) were measured. Furthermore, histopathological examinations were performed.

**Results:**

The results showed that ethanol induced gastric damage, improving oxidative stress markers level such as MDA (121 ± 4.45 nmol/mg proteins) and H_2_O_2_ (24.62 ± 1.04 μmol/mg proteins), increased pro-inflammatory cytokine (TNF-α level), as well as the expression of COX-2 in the ethanol group. However, a significant depletion of enzymatic and non-enzymatic antioxidants were observed, such as, GPx (72%), SOD (57.5%), CAT (41.6%) and -SH (50%). The lesions were associated with severe histopathological damage. The both *Citrus sinensis* peel aqueous extract (CSPE) and hesperidin significantly protect against all gastric damages caused by ethanol administration in rats.

**Conclusions:**

We propose that CSPE and hesperidin exhibit protective effects in EtOH-induced peptic ulcer in rat. This protection might be related in to part its antioxidant properties as well as its opposite effects on some studied intracellular mediators.

## Background

Gastric ulcers affect annually approximately 4 million people in the globe [1, 2]. This pathology is a heterogeneous disease of multifactorial etiology which can result from alcohol consumption, smoking, Helicobacter pylori infections, use of anti-inflammatory drugs (NSAIDs) [3]. This condition affects approximately 10-15% of the population in the world [4]. Gastric ulcers are typically caused by an imbalance between protective factors (blood flow, cell regeneration secretion, epithelial barrier and mucus secretion) and aggressive factors (reactive oxygen species [ROS] and acid-pepsin secretion) in the gastric mucosa [5]. The production of ROS plays a major role in the apparition and development of stomachal pathologies, such as gastric adenocarcinoma, peptic ulcerations, or gastritis [6]. In this respect, gastric mucosal layers represent a dynamic barrier in counteracting the effects of noxious agents through series of endogenous antioxidant defense systems. Indeed, this increase of oxidative damages is known to be related to the destructive factors-induced gastric mucosal damage [7, 8]. The mechanisms underlying the ethanol-induced gastric and duodenal injuries have not yet been fully elucidated.

Currently, the main pharmacological treatment for this disease is antisecretory drugs including, histamine type 2 receptor antagonists such as cimetidine and congeners, and irreversible proton pump inhibitors such as omeprazole, famotidine and congeners. Although effective, long-term treatment with these drugs is associated with several side effects and poor gastric healing, leading to ulcer recurrence [9].

The clinical importance of this pathology has led to the development of many pharmaceuticals and researches have already tested several natural compounds to prevent or treat gastric ulcers. Several naturally occurring antioxidant compounds were largely used to protect against gastric and duodenal diseases both in experimental and clinical situations. The plants and herbs are used to treat different diseases such as gastrointestinal illnesses, including peptic ulcers without side effects in ayurvedic medicinal system [10].

In the other hand, during fruit consumption’s, large quantities of wastes accumulate, however these materials may have some constituents of great significance generate substantial quantities of phenolics-rich sub products, which could be valuable natural sources of polyphenols. Hesperidin, is one of the bioflavonoids which is greatly found in Citrus species and is one of the major active constituent of tangerine (*Citrus reticulata*) and sweet orange (*Citrus sinensis*) peel [11, 12]. Many specific scientific efforts have demonstrated that dietary flavonoids have a variety of pharmacological properties, qy [13] and anti-ulcer [14] activities. In addition, the consumption of specific dietary flavonoids has been correlated with a reduced risk in the onset of some chronic and complex diseases, such as different types of cancer, asthma and cardiovascular disorders [15, 16]. One of the most important pharmacological aspects of flavonoids is considered to be the antioxidant and free radical scavenging activities [17]. In this regard, many recent studies have demonstrated the benefit of the use of *Citrus sinensis* peel aqueous extract and hesperidin in the treatment of patients suffering from venous leg ulcer and/or haemorrhoids [18]. Interestingly, orange peel is an interesting source of phenolic compounds, and in particular flavonoids such as hesperidin. Furthermore, Orange peel is also the primary waste fraction in the production of orange juice, although it has been used as a source of hesperidin because of its high concentration within this material [19].

Accordingly, the present study was designed to evaluate the putative gastroprotective role of the aqueous extract of *Citrus sinensis* peel (CSPE) and hesperidin (15 days) against oxidative stress induced by acute ethanol exposure and the mechanism involved in such protection.

## Methods

### Plant materials

The fresh fruit peel of orange (*Citrus sinensis L.*) was collected from North Est Tunisia (Mornag, Ben Arous, Tunisia) in December 2014. The identity of the plants was confirmed and deposit at the herbarium of the Higher Institute of Biotechnology of Beja and recognized at Tunisian Gene Bank (GNB 000600017).

#### Flavonoids extraction and isolation

Flavonoid extraction and isolation was determined. Briefly, dried peel of orange (850 g) was exhaustively extracted with 80% aqueous methanol (3× 4 L) which was concentrated under vacuum to yield 225 g of a viscous greenish residue.

#### Total phenolic content

Total phenolic content was determined by the colorimetric Folin-Cieucalteu method [20]. Briefly, 500 μL of the extract were added to 10 ml of water and 0.5 ml of Folin-Cieucalteu reagent. After 5 min, 8 ml of 7.5% sodium carbonate solution were added. The reaction mixture was kept in the dark for 2 h and its optical density was measured at 765 nm using a UV-visible spectrophotometer (Beckman DU 640B). Gallic acid was applied as standard, and the results were expressed as mg of gallic acid equivalents per gram of dry weight (mg GAE/g DW).

#### Total flavonoids determination

Total flavonoid content was determined by the AlCl_3_ colorimetric method [21]. Briefly, 1 mL of the sample was mixed with 1 mL of 2% aluminium chloride solution. After incubation for 15 min at room temperature, the optical density of the reaction mixture was evaluated at 430 nm. Quercetin was used as a citation standard and the total flavonoid content was expressed as mg of quercetin equivalent per gram of dry weight (mg QtE/g DW).

#### Free radical-scavenging activity on DPPH

The antioxidant capacity of MBSAE was performed using 2,2-diphenyl-1-picrylhydrazyl (DPPH) radical-scavenging activity as previously described by Grzegorczyk et al., [22].

Briefly, various concentrations of CSPE (50, 100, 200, 400, and 600 μg/mL) were added to 1 mL of 0.1 mM methanol solution of DPPH and incubated at 27 °C during 30 min. The optical density of the sample was quantified at 517 nm. DPPH radical-scavenging activity (RSA), expressed as a percentage, was estimated utilizing the following formula:

Quercetin was used as a reference molecule in the same concentration as the test extract.

$$ \boldsymbol{RSA}\ \left(\%\right)=\left(\left({\boldsymbol{A}}_{\boldsymbol{DPPH}}\boldsymbol{\hbox{-}}{\boldsymbol{A}}_{\boldsymbol{control}}\right)/{\boldsymbol{A}}_{\boldsymbol{DPPH}}\right)\Big)\times \boldsymbol{100} $$

All the analyses were executed in triplicate. The efficacy concentration 50 (EC50) value was determined as the concentration (in μg/mL) of the compound required to scavenge 50% of the DPPH radical.

#### Superoxide anion scavenging activity

The superoxide anion (O2^•-^) scavenging activity was performed according to Marklund and Marklund [23]. Briefly, 0.2 ml various concentrations of CSPE were added to 5.7 mL of 50 mM Tris-HCl buffer (pH 8.2). The mixture was incubated at 25 °C during10 min and then added to 0.1 ml of 6 Mm pyrogallol (dissolved in 10 mmol/L HCl). The absorbance of the reaction mixture was determined at 320 nm and the superoxide anion scavenging activity (SASA) was calculated using the following formula

$$ \boldsymbol{SASA}\ \left(\%\right)=\left[\boldsymbol{1}\boldsymbol{\hbox{-}}\left({\boldsymbol{A}}_{\boldsymbol{1}}\boldsymbol{\hbox{-}}{\boldsymbol{A}}_{\boldsymbol{2}}\right)/{\boldsymbol{A}}_{\boldsymbol{0}}\right]\times \boldsymbol{100} $$

A_0_ is the autoxidation rate of pyrogallol for control (the change of the absorbance), A_1_ is the oxidation rate of pyrogallol for samples, and A_2_ is the absorbance of the sample blank.

### Antiulcerogenic activity of CSPE

#### Animals and treatment

Adult male Wistar rats (weighing 220-240 g; housed five per cage) were purchased from Pasteur Institute of Tunis and used in accordance with the local ethic committee of Tunis University for use and care of animals in accordance with the NIH recommendations. They were provided with food (standard pellet diet- Badr Utique-TN) and water ad libitum and maintained in animal house at controlled temperature (22 ± 2 °C) with 12 h light-dark cycle. Experimental protocols were approved with the guidelines of the Ethical Committee of Science Faculty of Tunis, Tunisia. The test was performed in compliance with the Commission Directive 2000/32/EC and the OECD Guideline 474.

The rats were divided into seven groups of 10 animals each. Group 1 and 2 were served as control and had bidistilled water (5 mL/kg, *b.w.*, *p.o.*). Groups 3, 4 and 5 were pre-treated with various doses of CSPE (100, 200 and 400 mg/kg, *b.w., p.o.*), while group 6 and group 7 were pre-treated respectively with omeprazole (20 mg/kg, *b.w., p.o.*) and Hesperidin (50 mg/kg, *b.w., p.o.*). The period of pretreatment was 15 days.

Animals were fasted for 24 h before the last administration of CSPE or reference molecules. After 2 h, each animal, except those of groups 1 and 2, was intoxicated by acute administration of EtOH (4 g/kg, *b.w., p.o.*). Sixty min later, animals were sacrificed.

Blood was collected in heparinized tubes. After centrifugation at 3000 g during 15 min, plasma was treated for PSA determination.

#### Evaluation of gastric mucosal damage

The stomach of each animal were removed and opened along its greater curvature. The tissues were gently rinsed in NaCl 0.9%. Ulcer indexes were determined as the sum of the lengths of the whole gastric lesions (in mm^2^).

#### Gastric tumor necrosis factor alpha (TNF-α) assay

The TNF**-**α level was detected by an ELISA kit supplied by Quantikine, R&D system (USA) according to the method of Maskos et al., [24].

#### Real-time PCR

The expression of COX-2 was determined by real time PCR according to the method of Walch and Morris [25]. Total RNA was isolated from gastric tissue and mRNA was purified using RNeasy Purification Reagent (Qiagen, Valencia, CA), reverse transcribed into cDNA, and amplified by PCR. The reaction mixture was subjected to 40 cycles of PCR amplification as follows: denaturation at 95 °C for 1 min, annealing at 60 °C for 1 min and extension at 72 °C for 2 min. Semi-quantitation was performed using gel documentation system (Quantity one, Germany). Primer sequences for amplification of COX-2 are:

- Forward: 5 -GCAAATCCTTGCTGTTCCAATC-3
- Reverse: 5 -GGAGAAGGCTTCCCAGCTTTTG-3

And for β-actin as a house keeping gene we used the following primers:

- Forward 5 -GCC ATG TAC GTA GCC ATC CA-3
- Reverse 5-GAA CCG CTC ATT GCC GAT AG -3.

#### Histopathological analysis

Immediately after sacrifice, small pieces of stomach and duodenum were harvested and washed with ice cold saline. Tissue fragments were then fixed in a 10% neutral buffered formalin solution, embedded in paraffin and used for histopathological examination. 5 μm^.^ thick sections were cut, deparaffinized, hydrated and stained with hematoxylin and eosin (HE). The sections were examined in blind fashion for all treatments.

#### Stomach mucosa preparation

After the gastric lesions analyses, the stomach mucosa was rapidly excised and homogenized in phosphate buffer saline (KH_2_PO_4_/K2HPO_4_, 50 mM, pH 7.4) with Potter-Elvehjem homogenizer. After centrifugation at 10000 g for 10 min at 4 °C, supernatants were used for biochemical determination of protein, free iron, H_2_O_2_, calcium, SH- groups, MDA and antioxidant enzyme activities.

#### Lipid peroxidation measurement

Lipid peroxidation was determined by MDA measurement according to the double heating method [26]. Briefly, aliquots from gastric mucosa homogenates were mixed with BHT-TCA solution containing 1% BHT (*w*/*v*) dissolved in 20% TCA (*w*/*v*) and centrifuged at 1000 g for 5 min at 4 °C. Supernatant was blended with 0.5 N HCl and 120 mM TBA in 26 mM Tris and then heated at 80 °C for 10 min. After cooling, absorbance of the resulting chomophore was determined at 532 nm using a UV visible spectrophotometer (Beckman DU 640B). MDA levels were determined using an extinction coefficient for MDA-TBA complex of **1.56 × 10** ^**5**^ **M × 1 cm** ^**−1**^.

#### Plasma scavenging activities

The free radical scavenging activities of plasma was measured using the DPPH radical method according to Brand-Williams et al., [27]. Briefly, 100 μL of plasma sample were added to 2 ml of 2,2-diphenyl-1-picrylhydrazyl (DPPH) in methanol solution (100 mM). After incubation at 37 °C for 30 min, 1 mL of chloroform was added and the solution was centrifuged at 3000 *g* for 10 min. The absorbance of clear supernatant was then determined at 517 nm using spectrophotometer (Beckman DU 640B). DPPH solution was used as a control and the plasma scavenging activities (PSA), expressed in percentage, was calculated according to the following equation:

$$ \mathbf{PSA}\ \left(\%\right)=\mathbf{100}\times \Big({\mathbf{A}}_{\mathbf{517}}\left(\mathbf{control}\right)\times {\mathbf{A}}_{\mathbf{517}}\left(\mathbf{sample}\right)/{\mathbf{A}}_{\mathbf{517}}\left(\mathbf{control}\right). $$

#### Antioxidant enzyme activities

The activity of **SOD** was determined using modified epinephrine assays [28]. At alkaline pH, superoxide anion O^2•-^ causes the autoxidation of epinephrine to adrenochrome; while competing with this reaction, SOD decreased the adrenochrome formation. One unit of SOD is defined as the amount of the extract that inhibits the rate of adenochrome formation by 50%. Enzyme extract was added to 2 mL reaction mixture containing 10 μL of bovine catalase (0.4 U/μL), 20 μL of epinephrine (5 mg/mL) and 62.5 mM of sodium carbonate/bicarbonate buffer pH 10.2. Absorbance was recorded at 480 nm.

**CAT** activity was assayed by measuring the initial rate of H_2_O_2_ disappearance at 240 nm [29]. The reaction mixture contained 33 mM H_2_O_2_ in 50 mM phosphate buffer pH 7.0 and CAT activity was calculated using the extinction coefficient of 40 mM^−1^ cm^−1^ for H_2_O_2_.

The activity of **GPx** was quantified following the procedure of Flohé and Günzler, [30]. Briefly, 1 mL of reaction mixture containing 0.2 mL of gastric supernatant, 0.2 mL of phosphate buffer 0.1 M pH 7.4, 0.2 mL of GSH (4 mM) and 0.4 mL of H_2_O_2_ (5 mM). The mixture was incubated at 37 °C for 1 min and the reaction was stopped by the addition of 0.5 mL TCA (5%, *w*/*v*). After centrifugation at 1500 g for 5 min, aliquot (0.2 mL) from supernatant was combined with 0.5 mL of phosphate buffer 0.1 M pH 7.4 and 0.5 mL DTNB (10 mM) and absorbance was read at 412 nm. The activity of GPx was expressed as nmol of GSH consumed/min/mg protein.

#### Non-enzymatic antioxidants measurement

##### Thiol groups (−SH) determination

The total concentration of thiol groups (−SH) was performed according to Ellman’s method [31]. Briefly, homogenates of gastric mucosa were mixed with 800 μL of 0.25 M phosphate buffer (pH 8.2) and 100 μL of 20 mM EDTA, and the optical density was measured at 412 nm (A1). Then, 100 μL of 10 mM DTNB were added and incubated during 15 min and the absorbance of the sample was quantified at 412 nm (A2). The thiol groups concentration were calculated from A1 to A0 subtraction using a molar extinction coefficient of 13.6 × 10^3^ M^−1^ × cm^−1^. The results were expressed as μmol of thiol groups per mg of protein. GSH was estimated in gastric tissue by the method of Sedlak and Lindsay, [32]. Briefly 500 μL of tissue homogenate prepared in 20 mM EDTA, (pH 4.7) were mixed with 400 μL of cold distilled water and 100 μL of 50% TCA. The samples were shaken using vortex mixer and centrifuged at 1200×g during 15 min. Following centrifugation, 2 mL of supernatant were mixed with 400 μL of 400 mM Tris–buffer (pH 8.9) and 10 μL of 10 mM DTNB. The absorbance was read at 412 nm against blank tube without homogenate.

##### H_2_O_2_ determination

The gastric H_2_O_2_ levels were performed according to Dingeon et al., [33]. Briefly, the hydrogen peroxide reacts with p-hydroxybenzoic acid and 4-aminoantipyrine in the presence of peroxidase leading to the formation of quinoneimine that has a pink color detected at 505 nm.

##### Iron measurement

The gastric non-haem irons were measured colorimetrically using ferrozine as described by Leardi et al., [34]. Briefly, the iron dissociated from transferrin-iron complex by a solution of guanidine acetate was reduced by ascorbic acid and reacted with ferrozine leading to the formation of pink complex measured at 562 nm.

##### Calcium determination

The gastric calcium levels were performed using a colorimetric method according to Stern and Lewis, [35]. However at alkaline medium, calcium reacted with cresolphtalein leading to a colored complex measurable at 570 nm.

#### Protein determination

Protein concentrations were determined according to Hartree [36], as is a slight change of the Lowry method [37]. Serum albumin was used as standard.

### Statistical analysis

The data were analyzed by unpaired Student’s *t*-test and were expressed as means ± standard error of the mean (S.E.M.). The data are representative of 10 independent experiments. All statistical tests were two-tailed, and a *p* value of 0.05 or less was considered significant.

## Results

### Total polyphenols and flavonoids contents

The aqueous extract of *Citrus sinensis* peel (CSPE) was firstly investigated for their phenolic compouds contents. As shown in Table 1, the CSPE exhibited high levels of total phenolics (169.94 ± 2.13 mg gallic acid equivalence (GAE)/g dry matter (DM)] as well as total flavonoids (87.48 ± 1.59 mg quercetin equivalents (QtE)/g DM).

**Table 1:** Total polyphenols and flavonoids contents and IC50 values of the DPPH and superoxide anion free radicals scavenging assay of the CSPE and quercetin

	Total phenolic content (mg GAE/g)	Total flavonoids content (mg QE/g)	DPPH IC_50_ (μg/ml)	Superoxide anion IC_50_ (μg/ml)
CSPE Quercetin	169.94 ± 2.13 nt	87.48 ± 1.59 nt	188.49 61.4	198.4 126.83

### In vitro antioxidant capacity

Concerning the antioxidant capacity, we have found that the radical-scavenging activity of CSPE against superoxide anion and DPPH radicals increased significantly in a dose-dependent manner. The IC_50_ values corresponding to the amount of the fraction required to scavenge 50% of DPPH and O_2_^**·-**^ radicals are respectively 188.49 and 198.4 μg/mL. However, regarding quercetin, used as reference molecule, the IC_50_ values are 61.84 and 126.83 μg/mL, respectively for DPPH and O_2_^·-^ radicals (Table 1).

### Quantitative macroscopic evaluation of CSPE anti-ulcer activities

Concerning the macroscopic examinations, animals intoxicated with ethanol showed an extensive elongated thick, dark red and black band of hemorrhagic lesions on the glandular part of the stomach. Quantitative examination showed that CSPE, Hesperidin or reference drug pre-treatment significantly and dose-dependently reduced the ulcer index, and ameliorated the protection percentage of injury induced by EtOH administration (Table 2).

**Table 2:** Effect of *Citrus sinensis* peel extract (CSPE), Omeprazole (OM) and Hesperidin (H) on gastric macroscopic alterations induced by EtOH: ulcer index and Percentage of protection (%). Animals were pretreated with various doses of CSPE (100, 200 and 400 mg/kg, *p.o*.), OM (20 mg/kg, b.w*., p.o.*), H (50 mg/kg, *p.o*)

Pre-traitement	Stomach	
	Ulcer area (mm^2^) (Mean ± S.E.M)	Percentage of protection (%)
Control	0	--
EtOH	98.50 ± 3.59^*^	--
EtOH + CSPE-100	71.60 ± 1.9^**^	27.26**%**
EtOH + CSPE-200	47.60 ± 1.88^**^	53.19**%**
EtOH + CSPE-400	11.20 ± 0.86^**^	89.80**%**
EtOH + Omeprazole	21.34 ± 1.72^**^	78.16**%**
EtOH + Hesperidin	26.42 ± 1.74^**^	73.60**%**

The data are expressed as mean ± S.E.M. (*n* = 10) * *p* < 0.05 compared to control group and ^**^ *p* < 0.05 compared to EtOH group

### Histopathological evaluation of gastric lesions

**Fig. 1:**
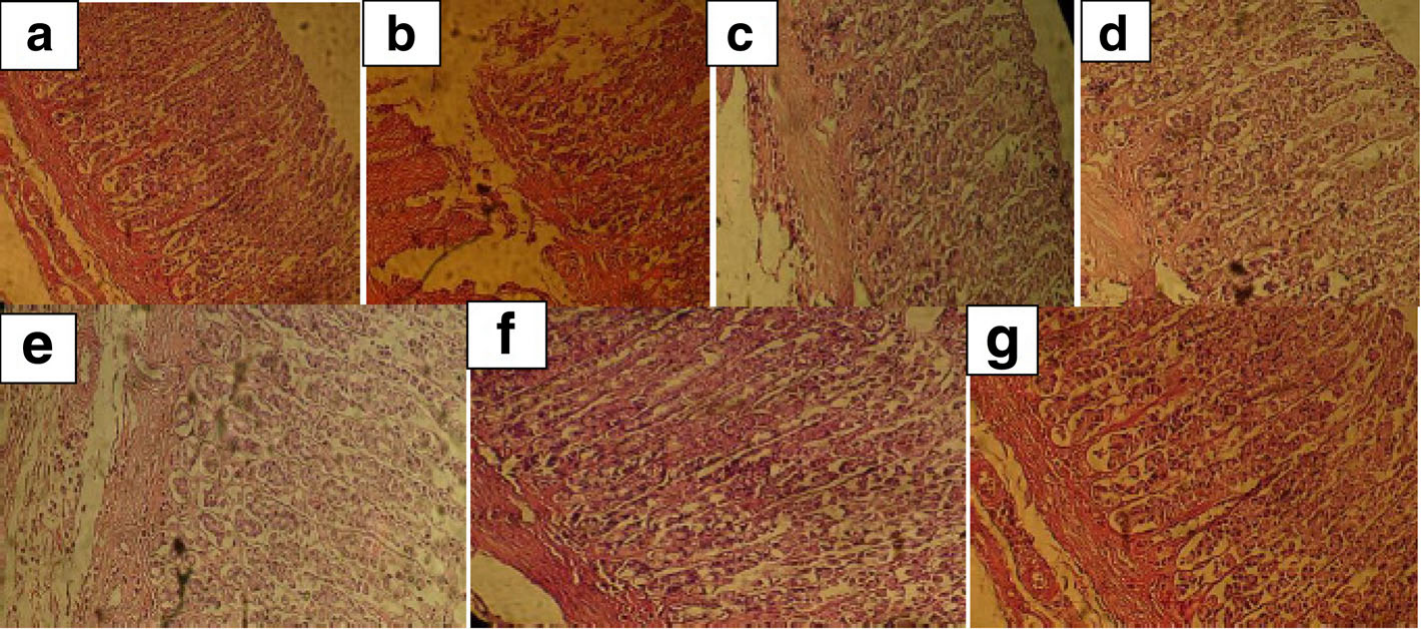
Gastric histology showing the protective effects of *Citrus sinensis* peel extract (CSPE), Hesperidin (H) and Omeprazole (OM) on EtOH-induced histological alteration in stomach. Animals were pre-treated with various doses of CSPE (100, 200 and 400 mg/kg, b.w., p.o.), OM (20 mg/kg, b.w., p.o.), Hesperidin (50 mg/kg, b.w., p.o) or bi-distilled water, challenged with a single oral administration of EtOH (4 g/kg, b.w., p.o.) or NaCl 9‰ for two hours. **a**: control; **b**: EtOH; **c**: EtOH+ CSPE-100; **d**: EtOH+ CSPE-200; **e**: EtOH+ CSPE-400 and **f**: EtOH+ H; **g**: EtOH+ OM). The data are expressed as mean ± S.E.M. (*n* = 10) *: *p* < 0.05 compared to the control group and **#**: *p* < 0.05 compared to the EtOH group

Histological observation of ethanol-induced gastric lesions in EtOH group showed a comparative extensive congestion, surface coating alteration, necrotic lesions, edema, epithelial and vascular cells alteration, haemorrhage and hyperaemia as well as inflammatory cell infiltration in the stomach (Fig. 1) mucosa and submucosa. Pretreatment with CSPE and Hesperidin or reference drug presents a clear dose-dependent protection of the gastric mucosa as seen by reduction of lesions, mucosal and submucosal edema as well as leucocytes infiltration. A similar protective effect had also observed in Hesperidin and omeprazole pre-treated rats.

### Gastric tumor necrosis factor alpha (TNF-α)

**Fig. 2:**
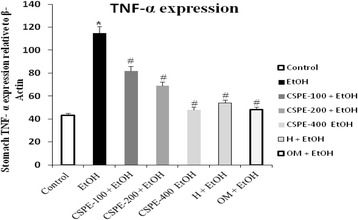
Subacute effect of *Citrus sinensis* peel extract (CSPE), Hesperidin (H) and Omeprazole (OM) on the concentration of gastric mucosal TNF-α on EtOH-induced ulcer in rats. Animals were pre-treated with various doses of CSPE (100, 200 and 400 mg/kg, b.w., p.o.), OM (20 mg/kg, b.w., p.o.), Hesperidin (50 mg/kg, b.w., p.o) or bi-distilled water, challenged with a single oral administration of EtOH (4 g/kg, b.w., p.o.) or NaCl 9‰ for two hours. The data are expressed as mean ± S.E.M. (*n* = 10) *: *p* < 0.05 compared to the control group and **#**: *p* < 0.05 compared to the EtOH group

Gastric TNF-α, a major pro-inflammatory cytokine, show any significant change in both and hesperidin and CSPE pretreated rats compared to untreated ulcer rats as shown in Fig. 2. A similar protective effect had also observed with Omeprazole-induced a significant reduction in gastric TNF-α compared to treat ulcerated rats (*P* < 0.05).

### Cyclooxygenase COX-2 gene expression

**Fig. 3:**
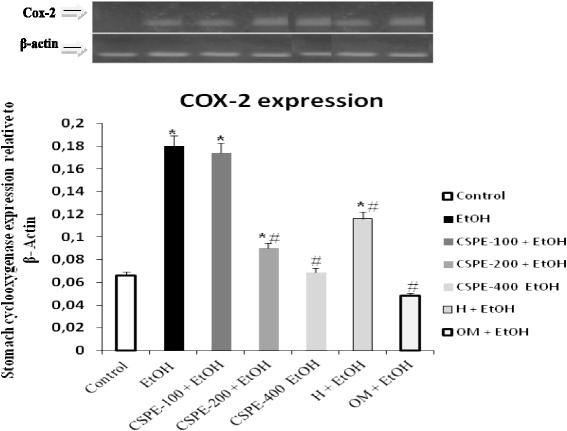
Subacute effect of *Citrus sinensis* peel extract (CSPE), Hesperidin (H) and Omeprazole (OM) on gene expression of **COX-2** relative to β-actin in gastric mucosa of rats induced by ethanol (EtOH) in rats. Animals were pre-treated with various doses of CSPE (100, 200 and 400 mg/kg, b.w., p.o.), OM (20 mg/kg, b.w., p.o.), Hesperidin (50 mg/kg, b.w., p.o) or bi-distilled water, challenged with a single oral administration of EtOH (4 g/kg, b.w., p.o.) or NaCl 9‰ for two hours. The data are expressed as mean ± S.E.M. (*n* = 10)*: *p* < 0.05 compared to the control group and **#**: *p* < 0.05 compared to the EtOH group

The real-time PCR assays showed that COX-2 mRNA was increased 2 h after EtOH administration in the rats stomach mucosa (*P* < 0.05). In addition, CSPE decreased COX-2 mRNA expression when compared to ulcerated untreated rats (Fig. 3), this decrease presents a clear dose-dependent. In addition, Omeprazole and hesperidin had significant effects on COX-2 mRNA expression when compared to ulcerated rats.

### Effects on plasma scavenging activity

**Fig. 4:**
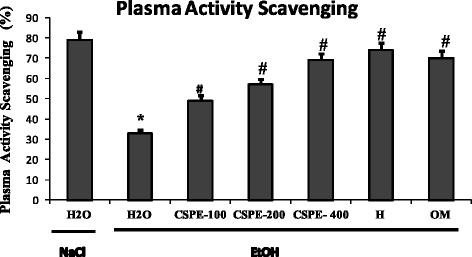
Subacute effect of *Citrus sinensis* peel extract (CSPE), Hesperidin (H) and Omeprazole (OM) on EtOH-induced disturbances in plasma scavenging activity (**PSA**). Animals were pre-treated with various doses of CSPE (100, 200 and 400 mg/kg, b.w., p.o.), OM (20 mg/kg, b.w., p.o.), Hesperidin (50 mg/kg, b.w., p.o) or bi-distilled water, challenged with a single oral administration of EtOH (4 g/kg, b.w., p.o.) or NaCl 9‰ for two hours. The data are expressed as mean ± S.E.M. (*n* = 10)*: *p* < 0.05 compared to the control group and **#**: *p* < 0.05 compared to the EtOH group

EtOH administration significantly decreased the plasma scavenging activity as compared to control group (Fig. 4). By contrast, PSA percentage was significantly and dose-dependently increased after CSPE pre-treatment. Similar effects were also observed for omeprazole and hesperidin, used as reference molecules.

### Effect of CSPE and hesperidin on EtOH-induced gastric lipoperoxidation and hydrogen peroxide increase

**Fig. 5:**
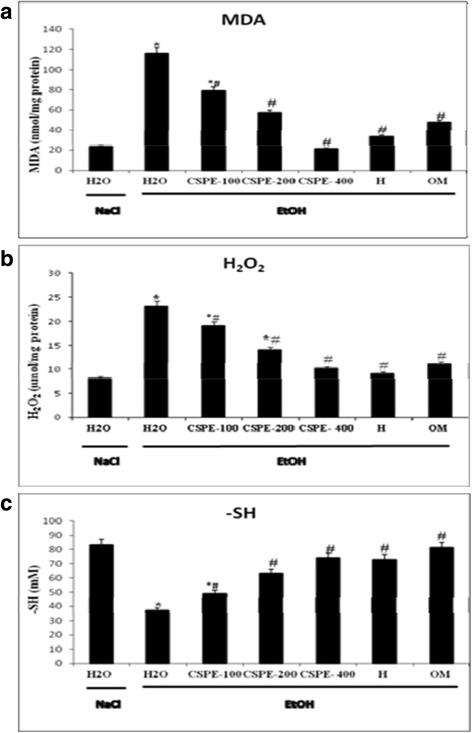
Subacute effect of *Citrus sinensis* peel extract (CSPE), Hesperidin (H) and Omeprazole (OM) and ethanol (EtOH)-induced changes in stomach mucosa on MDA (**a**), H_2_O_2_ (**b**) and –SH (**c**) levels in rats induced by ethanol (EtOH) in rats. Animals were pre-treated with various doses of CSPE (100, 200 and 400 mg/kg, b.w., p.o.), OM (20 mg/kg, b.w., p.o.), Hesperidin (50 mg/kg, b.w., p.o) or bi-distilled water, challenged with a single oral administration of EtOH (4 g/kg, b.w., p.o.) or NaCl 9‰ for two hours. The data are expressed as mean ± S.E.M. (*n* = 10) *: *p* < 0.05 compared to the control group and **#**: *p* < 0.05 compared to the EtOH group

Bearing on the effect of EtOH, CSPE and hesperidin on oxidative stress condition, we firstly studied the gastric lipoperoxidation and hydrogen peroxide content (Fig. 5). EtOH intoxication drastically increased the gastric MDA and H_2_O_2_ levels (Fig. 5a and b). CSPE pre-treatment significantly and dose dependently reversed lipoperoxidation and hydrogen peroxide increase induced by EtOH intoxication. The same results were observed with omeprazole and hesperidin pre-treated rats.

### Effect of CSPE and hesperidin on EtOH-induced gastric –SH groups decrease

We also showed that thiol group’s level was significantly reduced in the gastric mucosa of ethanol-treated rats. However, CSPE (100, 200 and 400 mg/kg, b.w. p.o.), hesperidin (50 mg/kg, b.w. p.o.) or omeprazole (20 mg/kg, b.w. p.o.) pre-treatment significantly protected against this decrease as compared to EtOH group (Fig. 5c).

### Effect of CSPE and hesperidin on EtOH-induced antioxidant enzyme activities depletion

**Fig. 6:**
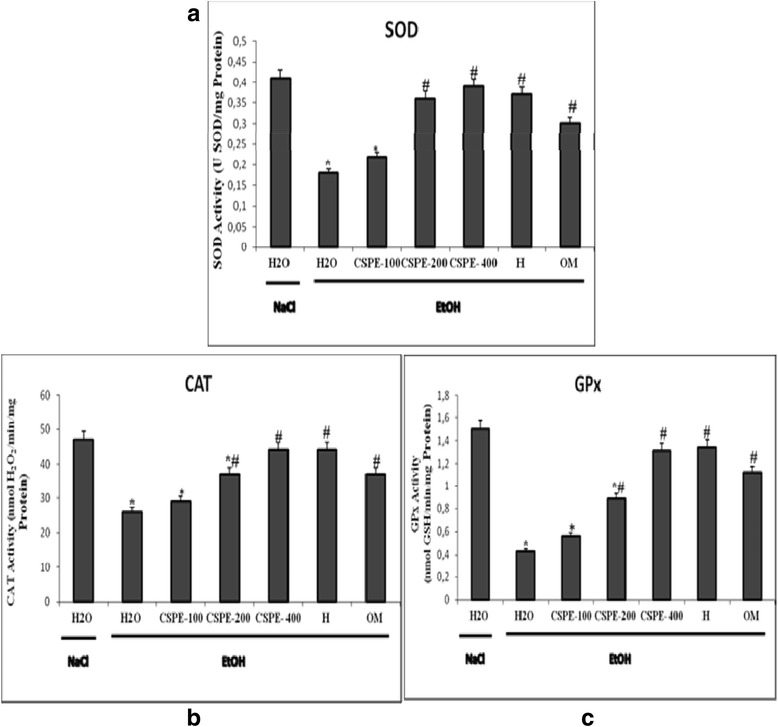
Subacute effect of *Citrus sinensis* peel extracts (CSPE), Hesperidin (H) and Omeprazole (OM) on ethanol (EtOH)-induced changes in stomach mucosa antioxidant enzyme activities: SOD (**a**), CAT (**b**) and GPx (**c**) in rats. Animals were pre-treated with various doses of CSPE (100, 200 and 400 mg/kg, b.w., p.o.), OM (20 mg/kg, b.w., p.o.), Hesperidin (50 mg/kg, b.w., p.o) or bi-distilled water, challenged with a single oral administration of EtOH (4 g/kg, b.w., p.o.) or NaCl 9‰ for two hours. The data are expressed as mean ± S.E.M. (*n* = 10)*: *p* < 0.05 compared to the control group and **#**: *p* < 0.05 compared to the EtOH group

We further looked at the effect of EtOH, CSPE and hesperidin on antioxidant enzymes activities in gastric mucosa (Fig. 6). Alcohol, significantly decreased antioxidant enzyme activities such as SOD (A), CAT (B) and GPx (C). However, subacute pre-treatment with *Citrus sinensis* peel aqueous extract, hesperidin and omeprazole significantly reduced the EtOH-induced decrease in antioxidant status to near control levels especially with the highest dose of CSPE 400 mg/kg b.w.

### Effects on free iron and calcium

Bearing on the effect of EtOH, CSPE and hesperidin on intracellular mediators such as free iron and calcium levels in gastric mucosa (Table 3). Ethanol group showed a significant decrease in free iron and ionizable calcium levels in gastric tissues when compared to control group. However, the *Citrus sinensis* peel aqueous extract (CSPE) treatment significantly and dose-dependently reduced the EtOH-induced intracellular mediators deregulation. A similar protective effect had also observed in Hesperidin (H) and omeprazole (OM) in pre-treated rats.

**Table 3:** Effect of *Citrus sinensis* peel extract (CSPE), Hespiridin (H) and Omeprazole (OM) on EtOH-induced disturbance in gastric H_2_O_2_, free iron and calcium levels. Animals were pretreated with various doses of CSPE (100, 200 and 400 mg/kg, *p.o*.), OM (20 mg/kg, b.w., *p.o.*), H (50 mg/kg, p.o) or NaCl 9‰

Pretreatment	Free iron (μmol/mg protein)	Calcium (mmol/mg protein)
Control	18.08 ± 1.06	101.69 ± 6.91
EtOH	42.14 ± 2.43^*^	194.18 ± 16.13^*^
EtOH + CSPE-100	33.36 ± 1.38^**^	142.64 ± 9.46^**^
EtOH + CSPE-200	26.12 ± 1.17^**^	129.88 ± 7.68^**^
EtOH + CSPE-400	19.68 ± 1.53^**^	113.99 ± 9.19^**^
EtOH +Omeprazole	22.29 ± 1.67^**^	117.44 ± 14.27^**^
EtOH + Hesperidin	20.76 ± 1.20^**^	104.38 ± 13.26^**^

The data are expressed as mean ± S.E.M. (*n* = 10) * *p* < 0.05 compared to control group and ^**^ *p* < 0.05 compared to EtOH group

## Discussion

Medicinal plants are sources of some well known anti-ulcer drugs such as: liquorice (dehydroglycerrizinic acid), *Piper betel* L. (piperine), *Emblica officinalis* (embline), *Terminalia bellerica* and *Terminalia chebula* mostly in the category of glycosides and flavonoids [38]. Hesperidin is a flavanone glycoside abundant in orange peel especially *Citrus sinensis* and is an economical by-product of citrus. The present study was designed to investigate the anti-ulcer activity of *Citrus sinensis* peel aqueous extract and hesperidin isolated from *C. sinensis* on oxidative damage-induced gastric ulcer models, looking into its potential antioxidant and anti-inflammatory properties.

In vitro, our phytochemical study firstly showed that CSPE presents a powerful scavenging action against DPPH radical and superoxide anion with lower IC_50_ values (188.49 and 198.4 μg/mL, respectively). However, similar free radical-scavenging activities were previously observed for other plant extracts but lesser than CSPE [39, 40]. The antioxidant activity of CSPE could be, in part, attributed to its high phenolic compounds levels. In this context, our data also suggest that CSPE presents a high concentration of total polyphenols (169.94 ± 2.13 mg GAE/g), flavonoids (87.48 ± 1.59 mg QE/g). This antioxidant capacity of *Citrus sinensis* peel aqueous extract is mainly related to the higher level of phenolic compouds in this fraction [41]. However, these molecules are the major source of their capacity of scavenging free radicals such as superoxide anion (O_2_^.^) and hydroxyl radical (OH^.^) [42].

In vivo, we firstly showed that alcohol administration clearly altered the gastric mucosa and submucosa. These lesions are accompanied by edema, epithelial and vascular cells alteration, necrosis and leucocytes infiltration of the submucosal layer. Our data are in line with previous report using EtOH as ulceration inducer [43, 44]. Several mechanisms are implicated in the development of alcohol-induced lesions in the mucous membranes [45]. Ethanol is considered one of the agents that induce gastric ulcers. The effects of ethanol on gastric mucosa are complicated and multifaceted that may be associated with a disturbance in the balance between gastric mucosal protective and aggressive factors [46]. Ethanol causes injures in the vascular endothelial cells of the gastric mucosa and induces microcirculatory disturbance and hypoxia, linking to the overproduction of oxygen radicals [47]. ROS are produced within the gastrointestinal tract, but their roles in pathophysiology and disease pathogenesis have not been well studied.

Subacute treatment with CSPE protects against gastric lesions induced by EtOH administration and allowed to the reduction of morphological and histopathological observed signs. This therapeutic effect, using CSPE high dosage (400 mg/kg b.w; *o.p*), is more effective than reference molecules such as omeprazole (78.16 and 80.20%) and hesperidin (73.60 and 81.45%), respectively for the stomach mucosa.

Gastric TNF-α, a major pro-inflammatory cytokine, show a significant change in both CSPE and hesperidin treated rats compared to untreated ulcer rats as shown in Fig. 2. Omeprazole induced a significant reduction in gastric TNF-α compared to untreat ulcerated rats (*P* < 0.05).

The real-time PCR assays showed that COX-2 mRNA was increased 2 h after EtOH administration in the mucosa of the rats (*P* < 0.05). In addition, CSPE especially the higher dose and hesperidin decreased COX-2 mRNA expression when compared to ulcerated untreated rats (Fig. 5). The same effect was observed after omeprazole administration when compared to untreated ulcerated rats.

We also showed in the present study that EtOH intoxication induced an increase of lhe final products of lipid peroxidation, decrease of thiol group levels, increase of hydrogen peroxide content as well as depletion antioxidant enzyme activities such as GPx, CAT and SOD. Acute alcohol-induced oxidative stress was commonly documented in gastric mucosa [48], liver [49], kidney [50], heart [51] and brain [52]. Ethanol administration provoked oxidative imbalance through a number of pathways including the generation of reactive oxygen species [53]. Lipid peroxidation level is an indicator of the generation of ROS in the tissue. However, SOD converts the reactive superoxide radical to H_2_O_2_, which was decreased in the gastric mucosa and if not scavenged by CAT, it can by itself cause lipid peroxidation by generation of hydroxyl radical [54]. More importantly, we showed that *Citrus sinensis* peel and hesperidin pre-treatment abolished acute EtOH-induced oxidative stress in the gastric mucosa. These data fully corroborated all previously reported in vivo [55] and in vitro [56] antioxidant and anti-inflammatory properties of CSPE. These finding corroborate with previous study wich have reported that the aqueous extract of CSPE contains a good amount of total polyphenols, total flavonoïds and condensed tannins [57]. These molecules are the primal source of the antioxidant ability of this plant, by scavenging free radicals as hydroxyl radical (OH^•^) which is the major cause of lipid peroxidation [58]. In addition, it is well known that sulfhydryls are in part involved in gastric cytoprotection [59] and also in the maintain of mucosal barrier integrity and scavenge free radicals formed due to the action of noxious agents [60].

More importantly, this study showed an increase in gastric and duodenal ionizable calcium in response to oxidative stress induced by ethanol administration. This result corroborated several previous studies [61, 62]. However, we can now speculate that CSPE, exerts its beneficial effect by chelating free iron and scavenging H_2_O_2_ leading to calcium homeostasis. Our results also suggest that pretreatment with CSPE protects against overloading of cells of the gastric and duodenal mucosa by free iron and H_2_O_2_ induced by ethanol sub-acute exposure. Nevertheless, free iron and hydrogen peroxide are the two components of the Fenton’s reaction, which is involved in the generation of hydroxyl radical (OH^•^) [63]. However, this later is the most powerful oxidant that can attack the molecular structures and thus play a major role in oxidative damage [64]. Living organisms so, develop a complex endogenous and exogenous antioxidant defense system to block the production of this harmful radical [65].

## Conclusion

In conclusion, *Citrus sinensis* peel aqueous extract and its major flavonoid, hesperidin, pre-treatment protects against gastric ulcer induced by ethanol in rats. Both compounds decreased COX-2 expression and gastric DNA fragmentation. Both drugs have successfully reduced TNF-α production and corrected the increased gastric lipid peroxidation. Therefore, these extract and flavanone exert anti-inflammatory and antioxidant properties and it is preferable to administer them under various inflammatory conditions to avoid the aggravation of the ulcers.

## Abbreviations

CAT: Catalase
CSPE: *Citrus sinensis* peel extract
DPPH: 2,2-diphenyl-1-picrylhydrazyl
GPx: Glutathione peroxidase
H: Hesperidin
H_2_O_2_: Hydrogen peroxide
MDA: Malondialdehyde
OM: Omeprazole
SOD: Superoxide dismutase
